# Case report: First autochthonous *Babesia vulpes* infection in a dog from Italy

**DOI:** 10.3389/fvets.2025.1498721

**Published:** 2025-02-19

**Authors:** Maria Teresa Antognoni, Valentina Cremonini, Ambra Lisa Misia, Federica Gobbo, Federica Toniolo, Arianna Miglio

**Affiliations:** ^1^Department of Veterinary Medicine, University of Perugia, Perugia, Italy; ^2^Experimental Zooprophylactic Institute of Venice, Legnaro, Italy

**Keywords:** babesiosis, canine babesiosis, *Babesia vulpes*, dog, anemia, PCR

## Abstract

A 10-month-old intact female Cane Corso dog was referred to the Veterinary Teaching Hospital of the University of Perugia (PG-VTH) for severe hemolytic anemia and thrombocytopenia. The dog had never traveled abroad and was regularly treated with antiparasitic products. On physical examination, the dog showed lethargy, delayed growth, weight loss, pale mucous membranes, and abdominal pain. The temperature was normal, and on examination, no ectoparasites were observed on the animal’s body surface. The main laboratory findings were hemolytic anemia, thrombocytopenia, and elevated liver enzymes. *Babesia* infection was initially diagnosed by blood smear evaluation via May–Grünwald–Giemsa staining and then confirmed by real-time polymerase chain reaction analysis; further sequencing analysis attributed the infection to *Babesia vulpes* (*B. vulpes*). An initial treatment with imidocarb dipropionate was only partially effective, while resolution of the infection was reached afterward with a combination of Malarone^®^ and azithromycin therapy. To the authors’ knowledge, this report describes the first case of *B. vulpes* infection in a dog in Italy.

## 1 Introduction

Babesiosis is a global emerging tick-borne disease caused by protozoa that infect red blood cells of several wild and domestic animals worldwide, including humans (1–3). Based on the size and morphology within the red blood cells (merozoite forms), babesias are divided into two groups: large (3–5 μm) and small (1.5–2.5 μm) babesias. More than 100 different species of the genus *Babesia* have been identified, and some of these can infect dogs, potentially causing severe forms of hemolytic anemia (4). The species most frequently causing infection in dogs are *Babesia canis*, *B. vogeli*, and *B. rossi* for large *Babesia* and *B. gibsoni* and *B. conradae* for small *Babesia*. Recently, another species of small *Babesia* called *Babesia vulpes* has also been reported to have clinical relevance in dogs (5). Cases of canine *B. vulpes* infection have been reported in some European and non-European countries (6–8) but never in Italy (3, 5, 9, 10), where it has currently been detected only in foxes and wild boars (11, 12). In this report, we describe the first documented case of canine babesiosis caused by *B. vulpes* in Italy. Diagnosis was made by microscopic blood smear evaluation and real-time polymerase chain reaction (PCR) analysis with gene sequencing.

## 2 Case description

A 10-month-old intact female Cane Corso dog was referred to the Veterinary Teaching Hospital of the University of Perugia (PG-VTH) for severe anemia and thrombocytopenia lasting longer than 1 month. The referring veterinarian, suspecting immune-mediated hemolytic anemia since the Coombs test positivity, started treatment with prednisone (2 mg/kg q24h), mycophenolate mofetil (10 mg/kg q12h), doxycycline (10 mg/kg q24h), and pantoprazole (1 mg/kg q24h). Nevertheless, despite a blood transfusion and a positive initial response to therapy, the dog subsequently developed worsening anemia.

When referred to the PG-VTH, physical examination revealed lethargy, delayed growth, weight loss, pale mucous membranes, and abdominal pain. The body temperature was normal. The dog was regularly vaccinated, and the owner stated regular application of products against endo- and ectoparasites. On examination, no ectoparasites were observed on the animal’s body surface.

**Figure 1:**
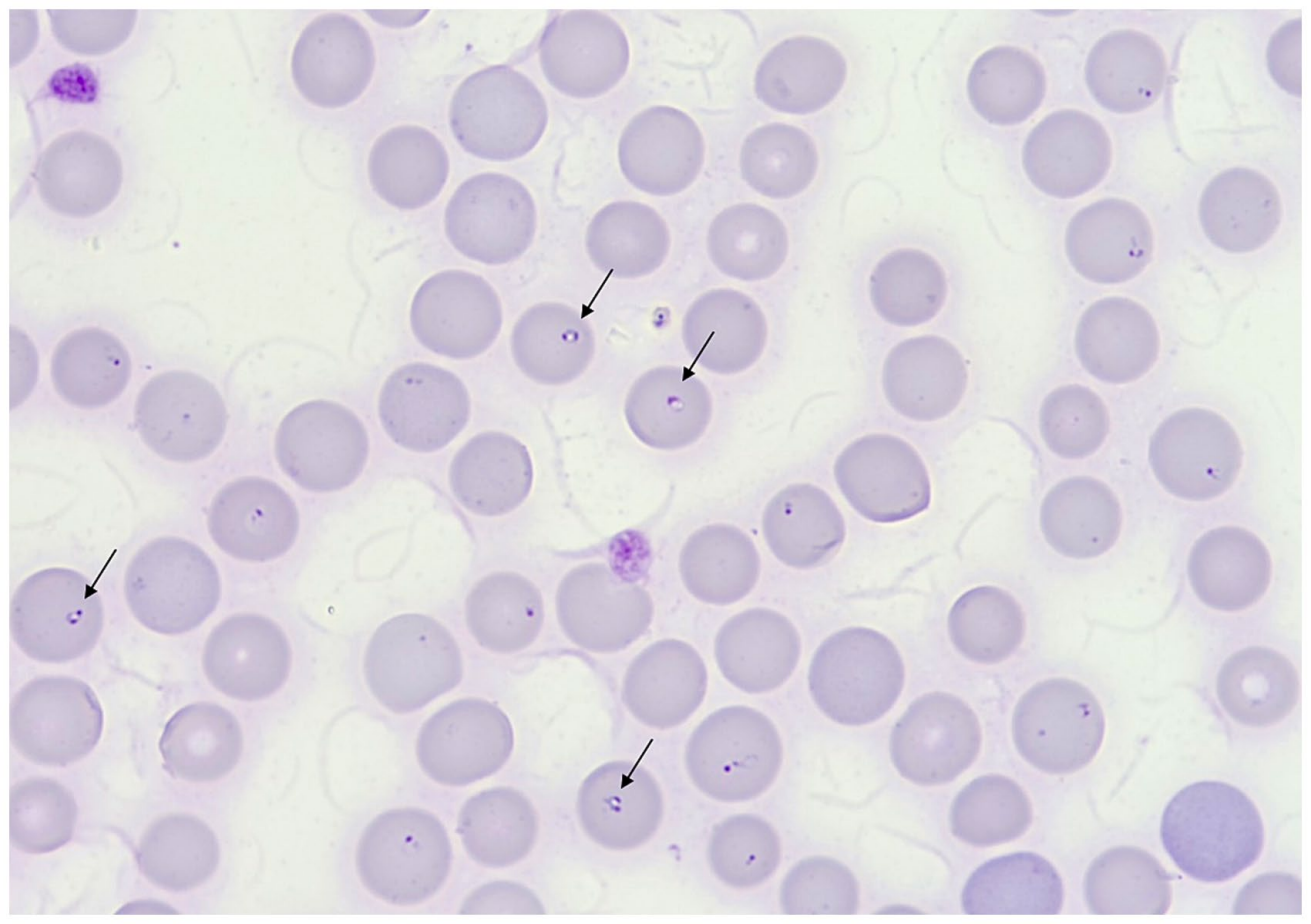
May–Grünwald–Giemsa stained peripheral blood smear showing anisocytosis and polychromasia. Black arrows indicate merozoites of *B. vulpes*. Original magnification 1,000×.

The complete blood count (CBC) revealed moderate macrocytic hypochromic anemia and severe thrombocytopenia (Sysmex XT 1800VET hematology analyzer, Sysmex, Kobe, Japan) (Table 1). On serum biochemical analysis performed on admission, increased concentrations of aspartate aminotransferase, alanine aminotransferase, alkaline phosphatase, and gamma-glutamyl transferase were present. Glucose and phosphorus concentrations were also mildly increased (Table 1) (Hitachi 904, Boehringer Mannheim, Germany). Abdominal ultrasonographic evaluation revealed no free abdominal fluid and no organ abnormalities. Multiple peripheral blood smears (PBS) were prepared and stained with May–Grünwald–Giemsa stain using an automatic slide stainer (Wescor Aerospray slide stainer, 7,120. Delcon, Bergamo, Italy). Slides were then observed under an optical microscope at 100× and 200× for cellularity assessment and then at 1,000× objective magnification for RBC morphology evaluation. The blood smear evaluation revealed a marked regenerative response and the presence of approximately 2 μm wide, intraerythrocytic, single to multiple signet ring-shaped organisms morphologically consistent with merozoites of small *Babesia* (Figure 1). The serological test (immunofluorescence antibody test, IFAT) for *Babesia* spp. was positive, while that for *Ehrlichia* spp., *Anaplasma* spp., *Rickettsia* spp., and *Leishmania infantum* was negative. A refrigerated ethylenediaminetetraacetic acid (EDTA) whole blood sample was sent to the Experimental Zooprophylactic Institute of Venice for real-time PCR analysis for *Anaplasma phagocytophilum*, *Ehrlichia canis*, *Rickettsia conorii*, and piroplasmids detection (*Babesia* spp., *Theileria* spp., and *Cytauxzoon* spp.).

**Table 1:** Complete blood count and biochemical parameters of the dog on admission at PG-VTH (day 0) and then during and after treatments (days 2 and 16: imidocarb dipropionate administration; day 135 to day 145: therapy with Malarone^®^ and azithromycin).

	Day 0	Day 15	Day 30	Day 75	Day 115	Day 245	Reference range
RBC (×10^6^/μL)	2.44	3.61	4.07	6.09	4.61	6.41	5.20–7.90
Hb (g/dL)	6.3	8.5	8.9	11.8	10.7	14.8	12.4–19.2
Hct (%)	22.7	27.4	27.5	34.5	33.2	42.9	35.0–52.0
MCV (fL)	93.0	75.9	67.6	56.7	72.0	66.9	60.0–71.0
MCH (pg)	25.8	23.5	21.9	19.4	23.2	23.1	21.9–26.3
MCHC (g/dL)	27.8	31.0	32.4	34.2	32.2	34.5	34.4–38.1
RDW (%)	18.2	17.6	18.3	18.8	15.1	14.7	13.2–19.1
WBC (×10^3^/μL)	15.98	10.86	14.43	10.83	14.63	10.72	5.60–17.80
Segmented neutrophils (×10^3^/μL)	13.06	8.57	10.79	8.21	11.03	7.22	2.90–13.60
Lymphocytes (×10^3^/μL)	1.99	1.56	2.06	1.66	2.74	2.44	1.10–5.30
Monocytes (×10^3^/μL)	0.69	0.71	1.50	0.58	0.45	0.36	0.40–1.60
Eosinophils (×10^3^/μL)	0.22	0.01	0.07	0.35	0.40	0.68	0.10–2.60
Basophils (×10^3^/μL)	0.02	0.01	0.01	0.03	0.01	0.02	0.00–0.05
PLT (×10^3^/μL)*	33	296	167	236	293	267	108–462
MPV (fL)	–	10.5	11.0	10.5	10.0	9.6	9.3–14.8
PCT (%)	–	0.31	0.18	0.25	0.29	0.26	0.12–0.60
PDW (%)	–	11.6	13.7	11.6	11.9	10.9	10.2–22.9
Total protein (g/dL)	6.9	–	–	6.5	–	7.1	6.0–8.5
Albumin (g/dL)	3.5	–	–	2.89	–	3.28	2.9–3.5
Creatinine (mg/dL)	0.89	–	–	1.13	–	1.35	1.0–2.0
Urea (mg/dL)	40	–	–	19	–	44	20–40
Glucose (mg/dL)	114	–	–	99	–	119	60–100
AST (IU/L)	91	–	–	34	–	34	9–40
ALT (IU/L)	150	–	–	33	–	35	7–40
ALP (IU/L)	646	–	–	106	–	85	10–100
GGT (IU/L)	48	–	–	4	–	6	<10
Total bilirubin (mg/dL)	0.36	–	–	–	–	–	0.07–0.71
Direct bilirubin (mg/dL)	0.16	–	–	–	–	–	–
Indirect bilirubin (mg/dL)	0.20	–	–	–	–	–	–
LDH (IU/L)	169	–	–	147	–	42	50–450
CPK (IU/L)	179	–	–	201	–	92	20–200
Calcium (mg/dL)	10.2	–	–	10.2	–	9.9	8.4–11.0
Phosphorus (mg/dL)	5.8	–	–	6.6	–	4.9	2.5–5.0

*All platelet counts were confirmed on peripheral blood smear examination.

RBC, red blood cell; Hb, hemoglobin; Hct, hematocrit; MCV, mean corpuscular volume; MCH, mean corpuscular hemoglobin; MCHC, mean corpuscular hemoglobin concentration; RDW, red blood cell distribution width; WBC, white blood cell; PLT, platelet; MPV, mean platelet volume; PCT, plateletcrit; PDW, platelet distribution width; AST, aspartate aminotransferase; ALT, alanine aminotransferase; ALP, alkaline phosphatase; GGT, gamma-glutamyl transferase; LDH, lactate dehydrogenase; CPK, creatine phosphokinase.

**Figure 2:**
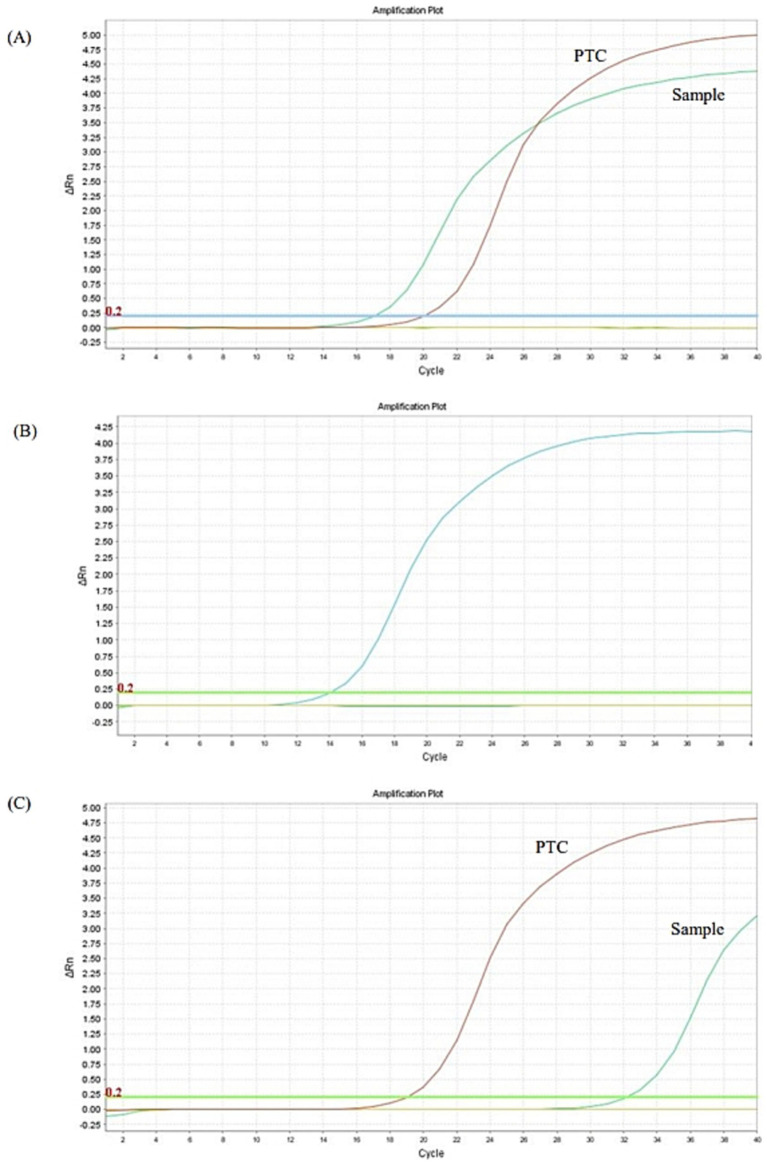
Real-time PCR analysis for *Babesia* spp. performed on the EDTA whole blood sample collected on days 0 **(A)**, 115 **(B)**, and 245 **(C)**. PTC, positive template control.

**Figure 3:**
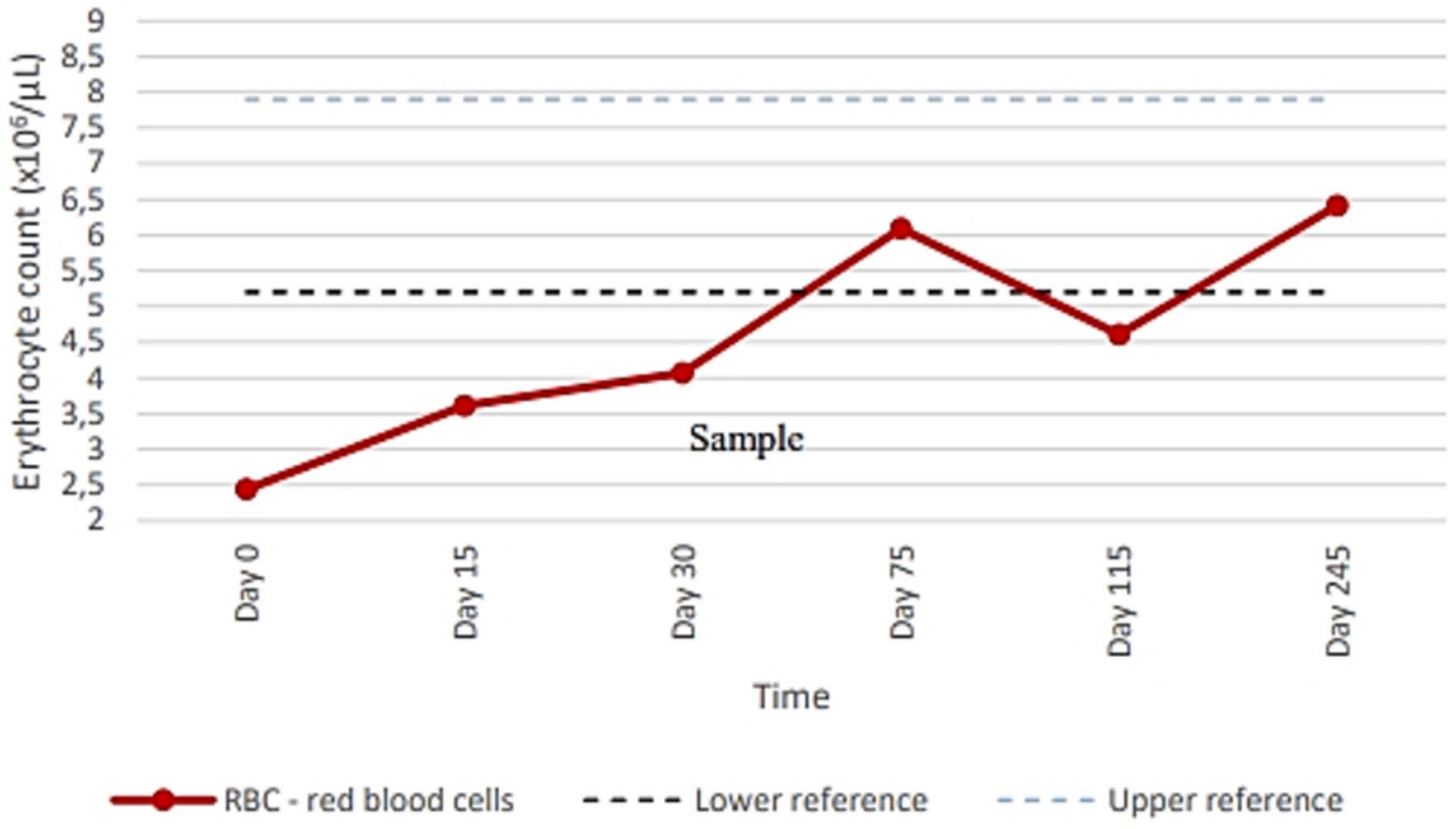
Erythrocyte counts at examined time points before, during, and after treatments. On days 2 and 16, imidocarb dipropionate administration was performed. From days 135 to 145, combined therapy with Malarone^®^ and azithromycin was administered.

The dog was hospitalized and a Dog Erythrocyte Antigen (DEA) 1 negative blood transfusion was administered. Pending the PCR analysis results, the first parenteral administration of antiprotozoal treatment with imidocarb dipropionate at 4.25 mg/kg was performed; all other previously prescribed therapies were maintained unchanged. The dog clinically improved and was discharged from hospital after few days. Real-time PCR analysis and gene sequencing then confirmed the diagnosis of babesiosis and identified *B. vulpes* (Figure 2A); the qualitative real-time PCR analyses for the detection of the other previously mentioned vector-borne pathogens were all negative. Considering the improvement in anemia and clinical conditions in the dog (Table 1; Figure 3: Day 15), the owners were reticent about changing therapy; so 2 weeks after the first administration of imidocarb dipropionate, it was decided to perform a second parenteral administration at the same dosage as the first, as reported by protocols (13, 14). Meanwhile, prednisone and mycophenolate mofetil doses were gradually decreased until they were discontinued. Over the following weeks, the dog showed progressive and encouraging improvement of anemia (Table 1; Figure 3: Days 30 and 75) and blood smear evaluation no longer revealed the presence of parasites.

Approximately 3 months after the discontinuation of the therapy, coinciding with the first heat of the dog, a new decrease in hematocrit was evidenced at CBC follow-up (Table 1; Figure 3: Day 115). On blood smear evaluation, intraerythrocytic merozoites were not observed, but a new EDTA whole blood sample was sent to the Experimental Zooprophylactic Institute of Venice for real-time PCR analysis, which still tested positive for *B. vulpes*, as suspected (Figure 2B). Based on these findings, therapy with Malarone^®^ (atovaquone and proguanil hydrochloride) (20 mg/kg q12h) and azithromycin (10 mg/kg q 24 h) was administered for 10 days, this being reported in literature as the elective therapy for the treatment of canine babesiosis due to small *Babesia* spp. infection (4, 15, 16). Subsequent CBC follow-up performed by the referring veterinarian showed a gradual improvement. Then, 3 months after the last treatment, the CBC follow-up performed at PG-VTH evidenced complete resolution of anemia (Table 1; Figure 3: Day 245), and the PCR analysis carried out at that time yielded a negative result (Figure 2C).

### 2.1 Molecular diagnostics

DNA was extracted from the EDTA blood sample using a High Pure PCR Template preparation Kit (Roche Diagnostics, Munich, Germany), according to the manufacturer’s instructions. The sample was screened for piroplasmids (*Babesia*/*Theileria*/*Cytauxzoon* spp.) using real-time PCR assay performed with primers and protocols previously described (17). The amplification reaction was carried out in a total volume of 20 μL containing 10 μL of QuantiNova SYBR Green PCR Master mix 2X (Qiagen GmbH, Hilden, Germany), 0.1 μM of forward and reverse primers, and 3 μL of extracted DNA. The thermal profile consisted of 2 min of activation at 95°C, followed by 40 cycles at 95°C for 5 s, 60°C for 30 s, and 60°C for 30 s. A melting curve analysis was then performed by slowly raising the temperature of the thermal chamber from 60 to 95°C. To identify the Piroplasmida species, the PCR product was purified and sequenced in both directions using the same forward and reverse primers as amplification primers in an ABI PRISM 3130 Genetic analyzer (Applied Biosystems, Foster City, CA, USA). Nucleotide sequences were compared with representative sequences available in GenBank database using the Basic Local Alignment Search Tool (BLAST). The sequence of the sample of the present study presented a query cover of 99%, a percent identity of 100%, and E-Value 0.0 with *Babesia vulpes* (accession number GenBank PQ270552). In addition, phylogenetic analysis was performed using MEGA X software, and a neighbor-joining tree was created using 22 18S rRNA gene sequences of recent isolates of *Babesia* spp., while one sequence of *Theileria equi* was included as the out-group (Supplementary Figure S1).

## 3 Discussion

*Babesia vulpes* is a small *Babesia* previously named *Babesia* “*microti-like*,” “*Babesia (Theileria) annae*,” “*Babesia cf microti*,” and *Babesia* “Spanish dog isolate” (6, 18). Protozoa of the genus *Babesia* are transmitted by ticks, but the specific vector of *B. vulpes* has not yet been clearly identified (19). It is assumed that the major vectors could be ticks of the genus *Ixodes*. In particular, *I. hexagonus* (also known as the hedgehog tick) is often suggested as the most likely primary vector in Spain, as endemic areas for *B. vulpes* closely correspond to its geographic distribution (20, 21). However, studies on transmission are lacking (5, 20, 22), and cases of *B. vulpes* infection have been reported in regions where *I. hexagonus* is not present (21, 23). Other suggested tick vectors are *Dermacentor reticulatus* (21, 24), *Ixodes ricinus* (25), *Ixodes canisuga*, and *Rhipicephalus sanguineus sensu lato* (5, 22), although there are no studies confirming their possible epidemiological role.

As regards canine babesiosis, additional transmission routes have been potentially included such as vertical transmission, direct transmission dog-to-dog (e.g., through biting), and transfusion of contaminated blood products (7, 21, 26).

*B. vulpes* is primarily responsible for asymptomatic infections in the red fox (*Vulpes vulpes*), which is considered the reservoir host and the main source of infection for domestic dogs (19, 27–29). Noteworthy, such spillover of parasites is fostered by the increasing urbanization process that leads to a closer contact between wildlife and domestic animal population (29–31).

The first report of canine babesiosis caused by *B. vulpes* has been described in Spain (32, 33), where the parasite has a high prevalence in the red fox population and is currently considered endemic in the north-west of the country (22). Sporadic cases of infection in dogs have also been reported, mainly as a single case report, in other European and non-European countries, such as Portugal (26), Croatia (34), Sweden (35), France (36), Serbia (37), Russia (7), Canada (6), North America (21, 38), and others (8, 39).

To the author’s knowledge, this is the first confirmed case of *B. vulpes* infection in a dog in Italy. Recently, the parasite has been identified in red foxes in Central Italy (12) and wild boars in Southern Italy (11), but it has never been reported in ticks or small domestic animals.

In this report, the dog had no history of traveling outside Italy or of being bitten by other dogs. Although owners stated that they have never observed ticks on the dog and reported a regular application of ectoparasite anti-feeding products, the transmission by an infected tick remains the strongest hypothesis. This is even more probable when considering that the dog was born in the region where Sgroi et al. (11) identified the parasite in wild boars and currently lives in a neighboring region to the one surveyed by Ebani et al. (12). Given the young age of the dog, vertical transmission could not be certainly ruled out. However, it was possible to identify the dog’s parents and siblings from the same litter, none of whom had shown signs of illness. In addition, the blood transfusion the dog received before being referred to the PG-VTH could also be considered a potential source of iatrogenic infection. Nevertheless, we are confident to exclude this way of transmission since the transfused blood product originated from our department’s veterinary blood bank (EMOVET-UNIPG), where animal donors are carefully selected by diagnostic screening of several blood-borne pathogens, including *Babesia* spp. (40, 41).

Canine babesiosis can cause a wide range of clinical signs, and severity of the disease primarily depends on the pathogenicity of the *Babesia* species and strain responsible for the infection (5). *B. vulpes* is reported to be associated with aspecific symptoms such as weight loss, weakness, fever, and anorexia or dysorexia. Moreover, hemolytic anemia, thrombocytopenia, and kidney failure are also described, with a substantial risk of death (7, 8, 16, 21, 26, 27, 42). However, in Spain, *B. vulpes* has also been identified in clinically healthy dogs, potential healthy carriers that by traveling may contribute to the spread of the infection (22). Nevertheless, cases of chronic asymptomatic infection are reported sporadically, and to date, *B. vulpes* is considered to be highly pathogenic (6), not least considering that the infection may lead to clinical disease even years later, as a consequence of immunosuppressive conditions developed by the infected animal (16, 22). The dog of this report showed aspecific clinical signs, and the main laboratory findings were hemolytic anemia, thrombocytopenia, and elevated liver enzymes, similar to other cases reported in literature (7, 16, 35). For the increased liver enzyme and glucose concentrations, a correlation with the steroid treatment the dog was receiving at the time of the examination was hypothesized. No evidence of renal impairment occurred either before or during the therapy, except for a mild increase in phosphorus concentration on admission. In contrast, other reported cases described dogs dying of severe renal failure, suggesting that azotemia is potentially associated with a poor prognosis in dogs infected by *B. vulpes* (7, 16, 35, 42). Thrombocytopenia, which is variously described in other reports (6, 7, 16), was severe but immediately improved during treatment with imidocarb dipropionate and immunosuppressive therapy. The anemia *status* was moderate to severe, macrocytic, and highly regenerative, and the Combs test performed gave a positive result. Immune-mediated hemolytic anemia (IMHA) is a frequent finding during *Babesia* infection (4), which is also reported in other cases sustained by *B. vulpes* (6, 16). Interestingly, in the case report described by Radyuk et al. (7), the dog showed a non-regenerative normochromic anemia, which the authors assumed to be a consequence of the renal impairment with decreased erythropoietin production.

In our case, microscopic evaluation of the blood smear was crucial for initial diagnosis of babesiosis. In fact, pleomorphic and ring-shaped small protozoan parasites (diameter of approximately 2 μm) were identified in approximately 20% of the erythrocytes. Subsequently, real-time PCR for Piroplasmida and further sequence analysis of the amplicon of PCR let us to attribute the blood infection to *B. vulpes*.

The resulting real-time PCR analysis and sequence analysis were essential for the setting of a target drug therapy that allowed the complete patient recovery. Thus, from a diagnostic point of view, it is important to apply molecular techniques capable of discriminating among *Babesia* species in affected dogs to evaluate accurately the clinical prognosis and the choice of the drug therapy. Indeed, after the administration of imidocarb dipropionate and a progressive improvement of clinical conditions and blood values, mild anemia relapse coincided with the dog’s first heat, 3 months later. This raised the suspicion of incomplete resolution of the small *Babesia* infection. At that time, although the dog showed no appreciable clinical signs and *Babesia* organisms were no longer visible at blood smear evaluation, a new sample of EDTA whole blood sample was therefore collected and again submitted to real-time PCR for Piroplasmida detection, which appeared to be still weakly positive for *B. vulpes*. It was then decided to administer the anti-*Babesia* treatment currently described for *B. gibsoni* and *B. vulpes* by Iguchi et al. (15) and Unterköfler et al. (16), respectively, consisting of Malarone^®^ (atovaquone and proguanil hydrochloride) and azithromycin continuously for 10 days. At subsequent follow-ups, the anemia improved to normal values and the real-time PCR for Piroplasmida was finally negative, confirming the effectiveness of this latter treatment. Noteworthy, a complete resolution of *B. vulpes* infection in dogs is rarely reported (6).

## 4 Conclusion

To the authors’ knowledge, this is the first case of *B. vulpes* infection in a dog in Italy.

Given the dog’s young age and considering that she had never traveled abroad, the most likely transmission routes could be the vertical one or *via* bites from infected ticks. The latter is supported chiefly considering that *B. vulpes* has recently been identified in wild animal species in regions of Italy, where the dog patient was born and has spent its life. Thus, *B. vulpes* infection should be considered in differential diagnosis in dogs with severe hemolytic anemia in Italy. In addition, the use of proper molecular analyses at the species level could reveal a parasite prevalence that has so far been underestimated. Furthermore, treatment with imidocarb dipropionate appears not to be completely effective, compared to the therapy with Malarone^®^ and azithromycin.

## Data Availability

The original contributions presented in the study are included in the article/Supplementary material, further inquiries can be directed to the corresponding author.

## Supplementary material

SUPPLEMENTARY FIGURE S1 Phylogenetic tree of 18S rRNA partial sequence of *B. vulpes* (Spanish dog), *B. vogeli*, *B. canis canis*, *B. gibsoni*, and *B. microti* using Neighbor-joining method. Each sequence is labeled with its accession number, name of the pathogen, year of sequence submission, host, and the geographic origin of the host. The tree is rooted using *Theileria equi* (AC: LC670595.1) as the out-group. Accession numbers of pooled sequences are *Babesia vulpes*: PQ270552.1, OQ816785.1, MW805763.1, PP232020.1, KY175162.1, MH699387.1, and MK742777.1; *Babesia Spanish dog*: AY534602.1 and EU583387.1; *Babesia vogeli*: OM948898.1 and OP169009.1; *Babesia canis canis*: MW588421.1 and HQ662634.1; *Babesia microti*: MN011934.1, JQ315422.1, MK986488.1, OR612069.1, MT423326.1, and PP087232.1; *Babesia gibsoni*: MN134514.1 and LC602469.1.
