# Supplementary figures and images for: Case report: First autochthonous Babesia vulpes infection in a dog from Italy
Source: Front Vet Sci. 2025 Feb 19;12:1498721. doi: 10.3389/fvets.2025.1498721 (PMC11880897; doi:10.3389/fvets.2025.1498721)

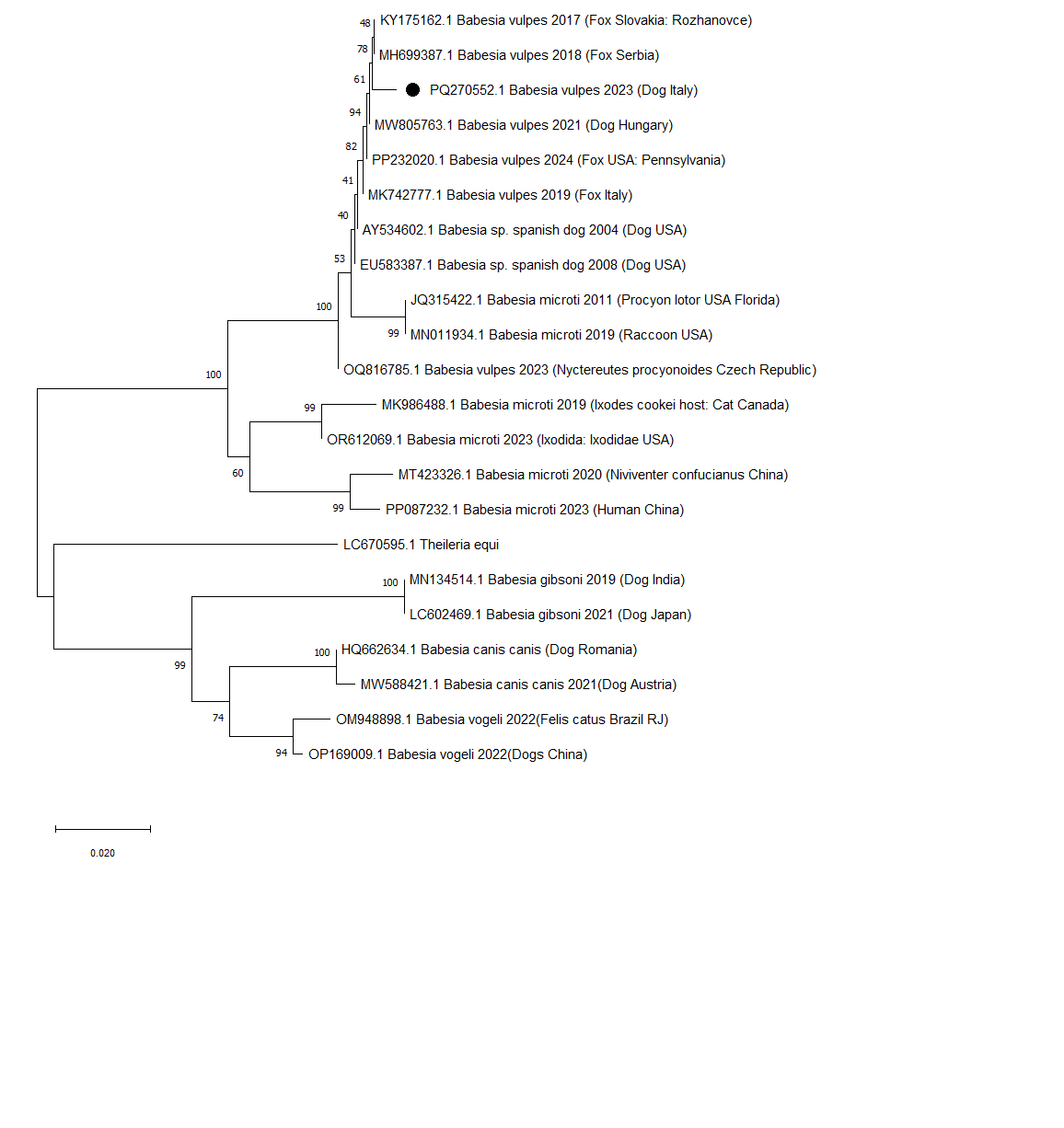

Supplement: SUPPLEMENTARY FIGURE S1 — Phylogenetic tree of 18S rRNA partial sequence of B. vulpes (Spanish dog), B. vogeli, B. canis canis, B. gibsoni, and B. microti using Neighbor-joining method. Each sequence is labeled with its accession number, name of the pathogen, year of sequence submission, host, and the geographic origin of the host. The tree is rooted using Theileria equi (AC: LC670595.1) as the out-group. Accession numbers of pooled sequences are Babesia vulpes: PQ270552.1, OQ816785.1, MW805763.1, PP232020.1, KY175162.1, MH699387.1, and MK742777.1; Babesia Spanish dog: AY534602.1 and EU583387.1; Babesia vogeli: OM948898.1 and OP169009.1; Babesia canis canis: MW588421.1 and HQ662634.1; Babesia microti: MN011934.1, JQ315422.1, MK986488.1, OR612069.1, MT423326.1, and PP087232.1; Babesia gibsoni: MN134514.1 and LC602469.1. [file Image_1.tif]
